# Assessing the limits of restraint-based 3D modeling of genomes and genomic domains

**DOI:** 10.1093/nar/gkv221

**Published:** 2015-03-23

**Authors:** Marie Trussart, François Serra, Davide Baù, Ivan Junier, Luís Serrano, Marc A. Marti-Renom

**Affiliations:** 1 EMBL/CRG Systems Biology Research Unit, Centre for Genomic Regulation (CRG), Barcelona, Spain; 2 Universitat Pompeu Fabra (UPF), Barcelona, Spain; 3 Gene Regulation, Stem Cells and Cancer Program, Centre for Genomic Regulation (CRG), Barcelona, Spain; 4 Genome Biology Group, Centre Nacional d'Anàlisi Genòmica (CNAG), Barcelona, Spain; 5 Institució Catalana de Recerca i Estudis Avançats (ICREA), Barcelona, Spain

## Abstract

Restraint-based modeling of genomes has been recently explored with the advent of Chromosome Conformation Capture (3C-based) experiments. We previously developed a reconstruction method to resolve the 3D architecture of both prokaryotic and eukaryotic genomes using 3C-based data. These models were congruent with fluorescent imaging validation. However, the limits of such methods have not systematically been assessed. Here we propose the first evaluation of a mean-field restraint-based reconstruction of genomes by considering diverse chromosome architectures and different levels of data noise and structural variability. The results show that: first, current scoring functions for 3D reconstruction correlate with the accuracy of the models; second, reconstructed models are robust to noise but sensitive to structural variability; third, the local structure organization of genomes, such as Topologically Associating Domains, results in more accurate models; fourth, to a certain extent, the models capture the intrinsic structural variability in the input matrices and fifth, the accuracy of the models can be *a priori* predicted by analyzing the properties of the interaction matrices. In summary, our work provides a systematic analysis of the limitations of a mean-field restrain-based method, which could be taken into consideration in further development of methods as well as their applications.

## INTRODUCTION

Recent studies of the three-dimensional (3D) conformation of genomes are revealing insights into the organization and the regulation of biological processes, such as gene expression regulation and replication (1–6). The advent of the so-called Chromosome Conformation Capture (3C) assays (7), which allowed identifying chromatin-looping interactions between pairs of loci, helped deciphering some of the key elements organizing the genomes. High-throughput derivations of genome-wide 3C-based assays were established with Hi-C technologies (8) for an unbiased identification of chromatin interactions. The resulting genome interaction matrices from Hi-C experiments have been extensively used for computationally analyzing the organization of genomes and genomic domains (5). In particular, a significant number of new approaches for modeling the 3D organization of genomes have recently flourished (9–14). The main goal of such approaches is to provide an accurate 3D representation of the bi-dimensional interaction matrices, which can then be more easily explored to extract biological insights. One type of methods for building 3D models from interaction matrices relies on the existence of a limited number of conformational states in the cell. Such methods are regarded as mean-field approaches and are able to capture, to a certain degree, the structural variability around these mean structures (15).

We recently developed a mean-field method for modeling 3D structures of genomes and genomic domains based on 3C interaction data (9). Our approach, called TADbit, was developed around the Integrative Modeling Platform (IMP, http://integrativemodeing.org), a general framework for restraint-based modeling of 3D bio-molecular structures (16). Briefly, our method uses chromatin interaction frequencies derived from experiments as a proxy of spatial proximity between the ligation products of the 3C libraries. Two fragments of DNA that interact with high frequency are dynamically placed close in space in our models while two fragments that do not interact as often will be kept apart. Our method has been successfully applied to model the structures of genomes and genomic domains in eukaryote and prokaryote organisms (17–19). In all of our studies, the final models were partially validated by assessing their accuracy using Fluorescence *in situ* hybridization imaging. However, no internal and systematic analysis of the accuracy of the resulting models has been performed and only an assessment of the reproducibility of these 3D reconstruction methods has been addressed (20).

Here, our main objective is to address the lack of such analysis by assessing the limits of 3D reconstruction based on mean-field restraint-based modeling. Although our analysis is based solely on models generated by TADbit, the conclusions are likely to hold for alternative mean-field restraint-based approaches. Over the next sections of the manuscript, we detail the methods for simulating ‘toy genome’ structures, deriving interaction matrices from them, reconstructing their 3D structure, assessing their quality and evaluating their accuracy using the Matrix Modeling Potential (MMP) score (Materials and Methods). Next, we describe the results of assessing the predictive power for determining the ‘real’ assembly structure of ‘toy genome’ structures as well as *a priori* evaluate the input interaction matrices modeling potential (Results). Finally, we summarize our conclusions on the limits of mean-field restraint-based approaches and how a measure such as the MMP can be used to *a priori* evaluate the reconstructed models (Discussion).

## MATERIALS AND METHODS

### Overall pipeline

**Figure 1.**
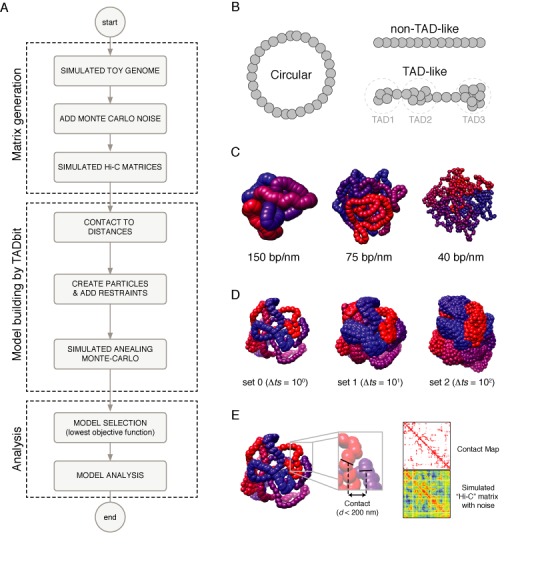
Matrix generation and model building. (**A**) Flowchart from toy genome generation to reconstructed 3D models. (**B**) Types of simulated genomic architectures. (**C**) Genomic density of simulated genomes. (**D**) Structural variability depending on the selection of conformations between distant time steps in the simulated genomes. (**E**) Derivation of interaction matrices from toy genome structures based on simulated restriction sites (black lines) and distance cut-off. Noise was added by a Monte-Carlo procedure with a probability proportional to the distance between the simulated restriction sites.

With the aim of assessing the accuracy of restraint-based modeling of genomes and genomic domains by TADbit (9,21), we devised a computational pipeline consisting of the following three steps (Figure 1A). First, using polymer modeling we simulated six artificially generated genomes (here called ‘toy genomes’) of a single chromosome with different architectures, from which we extracted 168 simulated interaction matrices with increasing noise levels and structural diversity. Second, we reconstructed with TADbit 3D models of the toy genomes based on their simulated ‘Hi-C’ interaction matrices. And third, we analyzed the reconstructed models for each simulation to assess their structural similarity to the original simulated toy genomes.

### Matrix generation from toy genome architectures

The toy genomes were generated using a worm-like chain (WLC) model, which provides a coarse-grained description of protein-coated DNA (e.g. the eukaryotic chromatin). At the ‘microscopic level’, a WLC is characterized by three parameters: the diameter (nm), the persistence length (nm) and the linear density (bp/nm), which respectively account for the physical thickness, the stiffness and the level of DNA compaction of the chain. Here, we considered a ‘chromatin fiber’ structure with a diameter of 30 nm and a persistence length of 100 nm, and investigated three densities: 40, 75 and 150 bp/nm. The toy genomes consisted of a single circular chromosome of ∼1 Mb long (Figure 1B) with a circular architecture to prevent the formation of knots during the WLC simulation. For half of the simulations, we forced into the toy genomes the formation of a Topologically Associated Domain (TAD)-like architecture by defining a limited number of locally interacting regions in the chromosome. To this end, we added a harmonic potential between all pairs of loci within the region considered as TAD so that they were constrained to remain close-by in space (22). Altogether, considering the combination of the three linear densities and the architectural properties (TAD or non-TAD), we investigated six types of genome architectures. Using a Monte-Carlo algorithm (23), we then simulated the equilibrium folding of these chromosomes in a cube of side 400 nm, which leads to the typical DNA density that is found in eukaryotic nucleus (0.015 bp*nm^−3^).

Each of the six simulations generated many successive conformations of the chromosomes, whose likelihood is dictated by thermodynamic laws (24). Using the outcome of these simulations, we generated simulated Hi-C matrices as explained below. To this end, each spatial conformation of the toy genome was segmented into *N* spherical bins of equal lengths, which determined the resolution of the Hi-C matrix. Given the ∼1 Mb length of our simulated chromosomes, we respectively considered bins of length 1.6 kb (626 bins), 2.5 kb (402 bins) and 5 Kb (202 bins) for the bp densities 40, 75 and 150 bp/nm, respectively (Figure 1C).

To assess the impact of cell-to-cell variability on our reconstruction method (25), we examined the effect of increasing the level of structural variability by selecting conformations of the toy genomes at different times of the simulations. For each of the six simulations (corresponding to the six chromosome architectures), we created a total of seven sets of 100 models, each differing in the number of simulation steps that separated them (Δ*t*) from 1 to 1 000 000 steps. The corresponding sets of toy genomes were named sets 0 to 6. The larger the Δ*t* between two selected models, the larger their structural variability (Figure 1D).

**Figure 2.**
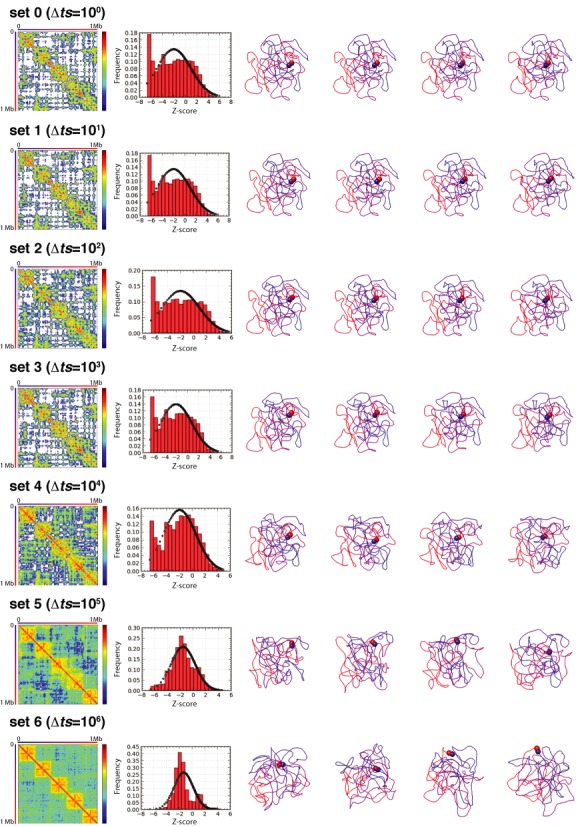
Simulated Hi-C interaction matrices. Simulated Hi-C interaction matrices for the toy genome architecture of chr75_TAD with noise levels α = 50. Each row shows the calculated matrix, the distribution of Z-scores and four randomly selected input structures, which are colored from particle 1 in blue to particle *N* in red, the start and end particles are highlighted with spheres. From top to bottom the figure depicts the simulated matrices from sets 0 to 6 ($\Delta t = 1\;{\rm to}\;\Delta t = 1\;000\;000$).

Finally, for each set of toy genome structures we derived an interaction matrix to obtain a ‘simulated Hi-C matrix’ by computationally mimicking the published Hi-C protocol (8) (Figure 1E). We set a restriction enzyme cutting frequency and defined all restriction site positions that would be tested for interactions (i.e. contact in the models). We considered ∼2000 restriction sites over the 1 Mb toy genome, which resulted in an average cutting frequency of 500 bp. We selected this frequency to consider it a middle range value of the restriction site frequencies used in the Hi-C experiments (26). Restriction enzymes recognizing a 6-base-pair sequence (e.g. HindIII) have an approximate cutting frequency of 4 Kb in the Human genome, while restriction enzymes recognizing a 4-base-pair sequence cut on average every 256 base pairs. The genomic position of each restriction site was determined randomly, maintaining the defined cutting frequency of 500 bp per genome. Once the restriction sites were assigned, we interpolated its 3D coordinates in the simulated toy genomes to obtain Euclidean distances between all the restriction sites. Next, we applied a 200 nm distance cut-off to generate a contact map between all the restriction sites in a set of structures (Figure 1E); this cut-off can be viewed as a maximum size of protein macro-complexes that can lead to Hi-C interactions through formaldehyde cross-linking. In addition, since several steps of the Hi-C protocol may affect the detection of interacting fragments (e.g. inefficient formaldehyde cross-linking or inefficient digestion and/or re-ligation) (26), we simulated the experimental noise by selecting pairwise interactions with a probability defined by a Gaussian procedure with an α value varying from 50 to 200 in steps of 50. The α parameter is related to the decay of the Gaussian function between the probability of interactions and the Euclidean distance between the restriction sites. A large α of 200 will increase the total probability of interactions, while a smaller α of 50 will decrease it. The selection of the Gaussian procedure allowed for a large dynamical range of maps across the tested structural variability. The resulting interaction matrices, that is, our ‘simulated Hi-C matrices’, thus contain a varying proportion of noise compared to a direct contact map generated from the models (Figure 1E). Finally, the total number of interactions between restriction sites was then pooled into bins according to the linear density of the genome (see above). The simulated Hi-C matrices contain thus a varying degree of experimental noise (α from 50 to 200), which are then complemented by an increasing degree of structural variability (sets 0 to 6) representing cell-to-cell variability in a population of millions of cells of a typical Hi-C experiment (Figure 2).

Before building models using TADbit, the input matrices were normalized by first calculating the weight (*W_i,j_*) for each pair of interactions:

\begin{equation*} w_{i.j} = \frac{{\sum\nolimits_{i = 1}^N {\sum\nolimits_{j = 1}^N {M_{i.j} } } }}{{\sum\nolimits_{i = 1}^N {M_{i.j} \times \sum\nolimits_{j = 1}^N {M_{i.j} } } }}.i\;j \in \;1..{\rm N}\end{equation*}

where *M_i,j_* is the raw counts in the simulated interaction matrix between bins *i* and *j*. The normalized matrix resulted from the multiplication of *M_i,j_* by its weight *W_i,j_*, which corresponds to a single iteration of the iterative correction and eigenvector decomposition (ICE) normalization procedure (27). Next, a decimal logarithm transformation was applied to the normalized interactions and its *Zscore_i,j_* was computed for non-zero interaction cells in the matrix as:

\begin{equation*} Z{\rm score}_{i.j} = \frac{{\log _{10} (M_{i.j} \times w_{i.j} ) - \mu }}{\sigma }\end{equation*}

where the average *μ* and the standard deviation σ from the entire matrix were obtained as:

\begin{equation*} \begin{array}{*{20}c} {\mu = {\rm log}_{10} \left( {\frac{{\sum\nolimits_{i = 1}^N {\sum\nolimits_{j = 1}^N {M_{i.j} \times w_{i.j} } } }}{{N \times N}}} \right)\;{\rm and}\;} \\ {\sigma = \sqrt[2]{{\frac{{\sum\nolimits_{i = 1}^N {\sum\nolimits_{j = 1}^N {((M_{i.j} \times w_{i.j} ) - \mu )^2 } } }}{N}}}} \\ \end{array}\end{equation*}

The resulting Z-scored matrices were used as input for modeling with TADbit.

### Model building by TADbit

To build the 3D models of the genomes, we used the TADbit python library developed around the IMP, which involves the translation of the data into particles; the assignment of spatial restraints between them and the search for optimal solutions maximizing the satisfaction of the imposed restraints. Next, we describe the used of our modeling protocol, which has been previously detailed (9).

Briefly, 3D models in TADbit are defined by *N* particles determined by the resolution of the input interaction matrix. Each particle has an excluded volume defined as a sphere with a radius proportional to the number of base pairs in each particle. Here, we consider an inverse relationship between spatial distances and the corresponding frequencies of interactions. Given this assumption, TADbit transforms the frequencies of interactions into spatial restraints differently for consecutive and non-consecutive particles. Two consecutive particles are spatially restrained (that is, kept at an equilibrium distance) according to their occupancy, which corresponds to the sum of their radii. Non-consecutive particles are restrained based on empirically identified parameters that define a set of restraints, their distances and the forces applied to them. TADbit empirically identifies three optimal parameters using a grid search where a limited number of models are built for each set of parameters. The three parameters are: the proximal distance between two non-interacting particles, a lower-bound cut-off to define particles that do not interact frequently and an upper-bound cut-off defining particles that do interact frequently. The resulting models for each combination of parameters are then used to calculate a contact map to compare it to the input interaction matrix by calculating the Spearman correlation coefficient between the two matrices (here called IMPSCC). Thus, similarly to many restraint-based methods for 3D genome reconstruction, TADbit sampling aims at identifying a set of models that maximizes the similarity between the models contact map and the Hi-C interaction matrix. Once the optimal parameters are identified, restraints are applied to the particles. Pairs of particles with contact frequencies above the upper-bound threshold are restrained to be at a given equilibrium distance. Pairs of particles with contact frequencies below the lower-bound threshold are maintained further than an equilibrium distance. Finally, TADbit uses a Monte Carlo simulated annealing sampling procedure to identify a set of 3D models that best satisfy the imposed restraints.

### Model accuracy

We assessed the structure similarity between the original toy genome architecture sets and the reconstructed models by computing two different measures. First, the distance Root Mean Square Deviation (dRMSD) between the best-reconstructed model and each of the 100 original selected structures was calculated after optimal superimposition of their structures by:

\begin{equation*} {\rm dRMSD} = \sqrt {\sum\limits_i {\sum\limits_j {(O_{ij} - R_{ij} )^2 } } } \end{equation*}

where *O_ij_* and *R_ij_* are the distance vectors between particle *i* and *j* in the original structure and in the reconstructed model, respectively. The dRMSD is a measure that varies between 0, when the two structures are identical, and a large number, proportional to the size of the object measured, when the two structures are completely different. The maximum dRMSD depends on the size of the object and the number of particles compared. Therefore, the reconstructed models were scaled to have the same dimensions in the three axes as the toy structures before structural superimposing them. The scale factor was calculated as the average ratio between the maximum distances in x-, y- and z-axis of the reconstructed models and the toy structures. Second, a distance Spearman correlation coefficient (dSCC) between all pairwise distances of particles in the best-reconstructed model and the corresponding ones in each of the 100 original toy structures was calculated. The dSCC measure varies between −1.0 and 1.0 for comparisons where the distances perfectly anti-correlate or correlate, respectively. Therefore, a model with a dSCC of 1.0 indicates good accuracy regardless of the scale of the compared structure.

### MMP

With the aim of identifying *a priori* whether an interaction matrix has the potential of being use for modeling, we calculated from each of the 168 simulated Hi-C matrices three different measures: (i) the contribution of the significant eigenvectors (SEV) from the matrix, (ii) the skewness and (iii) the kurtosis of the distribution of Z-scores in the matrix.

The contribution of the SEV score was obtained by first calculating the eigenvectors of the interaction matrix and the percentage of contribution of their corresponding eigenvalues. Next, we randomized 100 times the interaction matrix by shuffling the cells in the matrix that are equidistant from the diagonal. This shuffling strategy preserved the expected exponential decay of interactions as we go from the diagonal to the anti-diagonal corners of the matrix. From the 100 randomized matrices, we also calculated their eigenvectors and the percentage of contribution of their corresponding eigenvalues. We then set as ‘SEV’ those with eigenvalues above the mean eigenvalue plus two standard deviations of the equivalent eigenvectors in the random set of matrices. The final SEV score was the sum of the differences of the contribution of eigenvalues of all SEV:

\begin{equation*} {\rm SEV} = \sum\limits_i {\rm ev_i - \overline {\rm rev_i } } \end{equation*}

where ${\rm ev}_i$ corresponds to the contribution of the eigenvalue of the SEV *i* in the interaction matrix and $\overline {{\rm rev}_i }$ is the average contribution of the eigenvalue of the same eigenvector in the randomized 100 interaction matrices. Overall, large SEV scores are indicative of good potential for modeling. Intuitively, they indicate the presence of specific contacts that are not just the results of a random conformation of the chromosome.

The other two descriptive statistics were calculated directly from the distribution of Z-scores in the Hi-C matrices. First, the skewness statistic (SK) assesses in a single measure whether a score is skewed toward the right or left tails of its distribution. The kurtosis statistic (KT) complements the interpretation of the skewness. For example, matrices with skewness close to zero may result from multi-modal distributions of Z-scores. In such cases, the distribution will result in large KT scores. Therefore, the SK score will indicate skewness of the matrix toward positive or negative Z-scores and the KT score will indicate whether a matrix results or not in single-peaked distribution of Z-scores. For optimal modeling in TADbit, we expect no skewness and a single peak in the Z-score distribution. Both the skewness and the KT statistic were estimated using the SciPy python library (http://www.scipy.org). The SK and KT are calculated as:

\begin{equation*} {\rm SK} = \frac{\sum \nolimits_{i=1}^{N}(x_{i}-{\bar x})^3}{\sum \nolimits_{i=1}^{N}(x_{i}-{\bar x})^{2^{3/2}}}\quad {\rm KT} = \frac{\sum \nolimits_{i=1}^{N}(x_{i}-{\bar x})^4}{\sum \nolimits_{i=1}^{N}(x_{i}-{\bar x})^{2^{2}}} \end{equation*}

where *N* is the number of bins in the Z-score distribution and *x_i_* corresponds to the frequency of a given bin *i*.

Finally, to calculate the MMP score, we used the size (number of bins in the matrix), SEV, SK and KT for all 168 simulated Hi-C matrices as input to train a classifier with a linear regression kernel using Weka (28). During the training of the classifier, we used the actual accuracy of the produced 3D models (that is, the dSCC measure) as a target goal. We decided to use the dSCC measure instead of the dRMSD accuracy measure because it is independent of the scale and size of the objects to compare. The classifier, thus, aims at identifying a linear combination of the four matrix measures to produce a final score that best correlates with the dSCC of the models. We trained the classifier with a 10-fold cross-validation procedure, which resulted in a correlation coefficient of 0.84 between the MMP score and the dSCC measure. The MMP score is calculated as:

\begin{equation*} \begin{array}{*{20}c} {{\rm MMP} = - 0.0002*{\rm Size} + 0.0335*{\rm SK} - 0.0229*} \\ {{\rm KU} + 0.0069*{\rm SEV} + 0.8126} \\ \end{array}\end{equation*}

## RESULTS

### Toy genome structures and derived matrices

We investigated the reconstruction efficiency of six types of toy genomes hereafter labeled by ch40, ch75, ch150, ch40_TAD, ch75_TAD and ch150_TAD depending on the bp density along the chromosome and on the presence, or not, of TAD-like organization. To this end, for each toy genome, we generated seven sets of 100 different conformations, corresponding to seven different structural variability levels. More precisely, the nth set was generated by extracting 100 conformations separated by a time step of $\Delta t = 10^n$ iterations in the corresponding WLC simulation (Figure 2). Altogether, for each toy genome we generated 700 different chromosome conformations that were distributed among seven different sets, with set 0 having the lowest structural variability ($\Delta t = 1$) and set 6 the highest ($\Delta t = 10^6$). Such structural sets were then used to derive four contact maps with varying levels of experimental noise (that is, with α = 50, 100, 150 and 200), which simulate the results of a hypothetical Hi-C experiment. Finally, the contact maps were input to TADbit to build 3D models using a previously implemented protocol (9). The initial structural sets for the six tested toy genome architectures, their derived interaction matrices and the reconstructed 3D models are available at http://www.3DGenomes.org/datasets. Specific details on the construction of the toy genomes and the derived models are given in the Materials and Methods.

### Overall accuracy of the generated models

**Figure 3.**
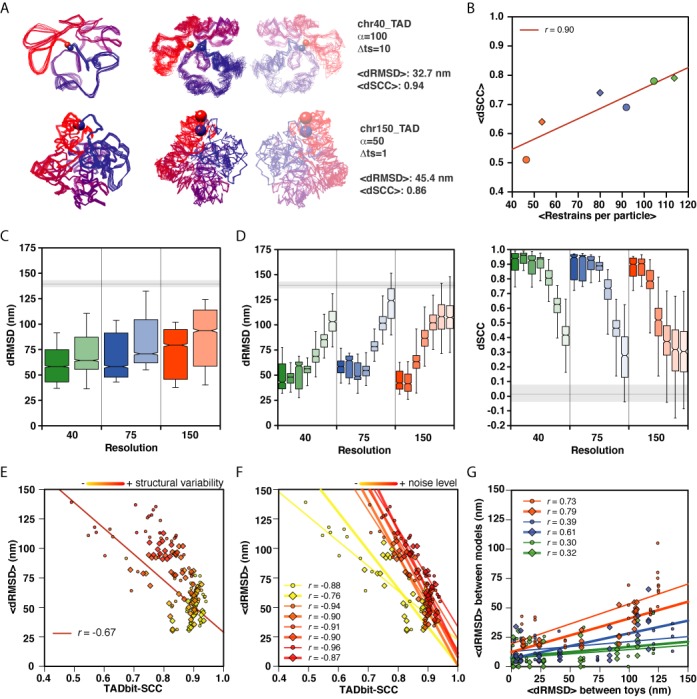
Model assessment. (**A**) Comparison of a 3D model ensemble of genome architectures for the chr40_TAD (top) and chr150_TAD (bottom) architectures. Superimposed input structures for set 0 (left models) and superimposed reconstructed 3D models (due to mirroring, TADbit generates right- and left-handed models (9)). Models are colored from particle 1 in blue to particle *N* in red, the start and end particles are highlighted with spheres. (**B**) Correlation between the restraints per particle and the accuracy of the reconstructed models as measured by the average dSCC score per architecture. Circle symbols correspond to non-TAD-like architectures. Rhomboid symbols correspond to TAD-like architecture. The colors indicate the toy genome density (green, blue and orange for 40, 75 and 150 bp/nm, respectively). (**C**) dRMSD distributions with respect to genome architecture. Colors correspond to the three density values with dark and pale colors corresponding to TAD-like and non-TAD-like architectures, respectively. Horizontal gray line and shade corresponds to the dRMSD distributing of comparing a ‘random genome’ of the same size and number of particles as the reconstructed models but with randomized coordinates. (**D**) Model accuracy as measured by dRMSD (left) and dSCC (right) with respect to the model density. Each density is colored as in panel A and contains seven distributions from the seven sets of structures from set 0 ($\Delta t = 1$) to high structural variability set 6 ($\Delta t = 10^6$) with dark to pale colors, respectively. Horizontal gray lines and shade as in panel C. (**E**) Correlation between the dRMSD values per reconstructed models and the Spearman correlation coefficient of the contact map from the reconstructed models and the original toy genome structures (TADbit- SCC). The points are colored proportional to the level of structural variability in the matrix (yellow to red from low set 0 ($\Delta t = 1$) to high structural variability set 6 ($\Delta t = 10^6$)). Shapes represented as in panel B. (**F**) Same as panel E but now the points are colored by the level of noise in the data (yellow to red for low to high levels of noise, that is from α = 50 to 200). The regression coefficients indicate the correlation per noise level α. (**G**) Correlation between structural variability in the toy genome structures and in the reconstructed models. Colors and shapes as in panel B.

To assess the accuracy of the genomic 3D models built by TADbit, we calculated two different accuracy measures between the reconstructed models and the toy genomic structures (that is, the dRMSD and the dSCC). Both measures of accuracy were calculated for all reconstructed models and averaged over architecturally similar toy genomes (Table 1). In total, we generated 168 simulated Hi-C matrices for the six toy genome architectures (that is, six architectures with seven levels of structural variability and each with four levels of noise in the data). The reconstructed architecture that best fitted the input structures corresponded to the 40 bp/nm density with a TAD-like architecture (chr40_TAD), with an average dRMSD of 60.5 nm and dSCC of 0.79. The architecture most difficult to reconstruct corresponded to 150 bp/nm density with no TAD-like features (chr150), with an average dRMSD of 86.4 nm and dSCC of 0.51. These values correspond to average measures over the 28 simulated Hi-C matrices per architecture, which include varying degrees of noise and structural variability. For example, within the chr40_TAD architecture, one of the best reconstructions corresponded to the matrix with mid noise level (α = 100), and low structural variability ($\Delta t = 10$), which resulted in a 3D model with dRMSD of 32.7 nm and dSCC of 0.94 (Figure 3A, top). Similarly, for the low-resolution architecture 150T, the best result (dRMSD = 45.4 nm and dSCC = 0.86) corresponded to a low level of noise (α = 50) and low structural variability ($\Delta t = 1$) (Figure 3A, bottom). In summary, TADbit was able to produce accurate models for all six toy genome architectures with a varying degree of accuracy depending on the levels of noise and structural variability in the simulated Hi-C matrices.

**Table 1.** Toy genome architectures and overall reconstruction accuracy

Name	Dens.(bp/nm)	TAD	Size	<Restraints per particle>	<Spearman CC>	<dRMSD>	<dSCC>
Chr40	40	no	626	104.4	0.84	71.12	0.78
Chr40_TAD	40	Yes	626	113.7	0.86	60.49	0.79
Chr75	75	no	402	91.8	0.84	82.14	0.69
Chr75_TAD	75	yes	402	79.9	0.86	68.56	0.74
Chr150	150	no	202	46.3	0.82	86.42	0.51
Chr150_TAD	150	yes	202	53.5	0.86	72.63	0.64

### Genome architecture and model accuracy

We tested two features of the toy genome architecture: its density (or resolution) and the presence or absence of local compact regions representing TADs. Models based on higher-resolution matrices resulted in a higher number of imposed restraints per particle in the reconstructed 3D models (Table 1). As expected, we observed a linear relationship between the number of restraints per particle imposed during modeling and the dSCC value (*r* = 0.9, Figure 3B), which in turn depends on the resolution of the input matrices determined by the density of the toy genomes. Despite the relative low accuracy of models for high-density genomes (i.e. low-resolution genomes), TADbit was able to generate topologies very similar to the input structures (Figure 3A). Altogether, these results indicate that the choice of genomic density and, with it, the resolution representing the genome alter the accuracy of the reconstructed models. The existence of a TAD-like organization in the genome had also an effect on the accuracy of the reconstructed models. All simulated matrices with genome architectures at 40 bp/nm density with TAD-like architecture resulted in an average dRMSD of 60.5 nm while genome architectures with no TADs resulted in an average dRMSD of 71.1 nm (Table 1). This trend was observed for all resolutions where the TAD-like architecture resulted in lower average dRMSDs (t-test *P*-value <0.001, Figure 3C). Overall, both high resolution simulated matrices and the existence of a TAD-like structures in the toy genomes resulted in more accurate reconstructed 3D models.

### The accuracy of the models is sensitive to structural variability but robust to noise

3C-based experiments are performed on tens of millions of cells and thus are a population-based interrogation of the genome. It is therefore likely that the interrogated cell population harbors structurally different conformations of their genome, due to the unsynchronized cell cycle or to natural cell-to-cell variability, among many other factors. To simulate such situation, we increased the structural variability in the input matrices by selecting structures from the architectural genomes at different simulation time steps (Materials and Methods). Simulated Hi-C matrices with increasing variability provided less detail of local chromosome structuring but captured the large-scale organization of the toy genomes such as the existence of TADs (Figure 2). As expected for any mean-field reconstruction method, the accuracy of our reconstructed genomes decreased with the increase in the input structural variability (Figure 3D and E). For all toy genomes with different architectures, the accuracy of the models was maintained up to the structural variability set 3 ($\Delta t = 1000$). The models resulting from the sparse matrices based on the structural sets 4 to 6 ($\Delta t \ge 10\;000)$ had significantly higher dRMSD values as compared to the other models. Indeed, model reconstruction based on low-resolution matrices (150 bp/nm genomes) and large structural variability resulted in models with poor accuracy (dRMSD > 90 nm). At the highest levels of structural variability (i.e. sets 4 to 6 or $\Delta t \ge 10\;000$), the interaction matrices were predominantly populated in the proximity of the diagonal, or the TAD structures, as the only common interacting regions between the different input structures for both the non-TAD-like and TAD-like architectures, respectively (see, for example, Figure 2, bottom rows). Interestingly, the reconstruction of 3D models with TADbit was robust to noise (Figure 3F). In fact, the accuracy of the models was constant to mid levels of noise in the data (average dRMSD of 70.7, 71.5, 74.3 and 78.7 for α values of 50, 100, 150 and 200, respectively). Nevertheless, the correlation between the TADbit-SCC and the dRMSD was higher at the mid level of noise compared to the low level of noise (0.77 for α = 50 and 0.87 for α > = 150). In summary, the reconstruction of 3D models based on noisy data is robust but mean-field methods are sensitive to structural variability in the simulated Hi-C interaction matrices.

### The TADbit-SCC is an accurate scoring function for modeling

TADbit model building depends on the imposed restraints for modeling, which in turn are determined by three optimized parameters. The three cut-offs are determined by maximizing the Spearman correlation coefficient between a contact map calculated from the reconstructed 3D models and the input simulated Hi-C matrix (here called TADbit-SCC). To test whether the TADbit-SCC measure is a good proxy for model accuracy, we compared it with the dRMSD of the resulting reconstructed genomes. The results clearly indicate that the use of the TADbit-SCC as a scoring function to identify the best models is reasonable (Figure 3E) as high values of TADbit-SCC are indicative of low dRMSD (*r* = −0.67). However, the relationship is not perfect and has two main properties that affect its adequacy for identifying good models: (i) a range of low dRMSD values may result in very similar TADbit-SCC and (ii) the dRMSD value saturates for low TADbit-SCC values. Altogether, the analysis indicates that the use of Spearman correlation coefficient (TADbit-SCC) as a scoring function during modeling is a good proxy for model accuracy but needs to be complemented by additional measures (see below).

### Reconstructed models capture part of the structural variability in the matrices

Mean-field restraint-based modeling methods assume that the interaction matrix reflects an average structure of the genome with a limited number of different conformations. Thus, such methods have intrinsic difficulties in capturing the variability of the data. To test whether our reconstructed models reflect the structural variability in the matrices, we calculated the dRMSD between the 100 input toy genome structures in each of the 168 matrices. We also calculated the dRMSD between 100 generated models per simulated matrix. In all the genomic architectures, we observed a correlation between the variability in the toy genome structures and the resulting variability in the reconstructed models (Figure 3G). The captured variability decreased with the increased number of restraints per particle (Figure 3B). That is, higher-resolution matrices that resulted in more restrained models have less structural variability in the output structures. Importantly, the degree of variability is ∼2-fold less in the resulting models compared to the input toy structures. Nevertheless, and despite the intrinsic limitations, the resulting models capture part of the structural variability in the matrices.

### Statistics of the input matrices correlate with the accuracy of the models

**Figure 4.**
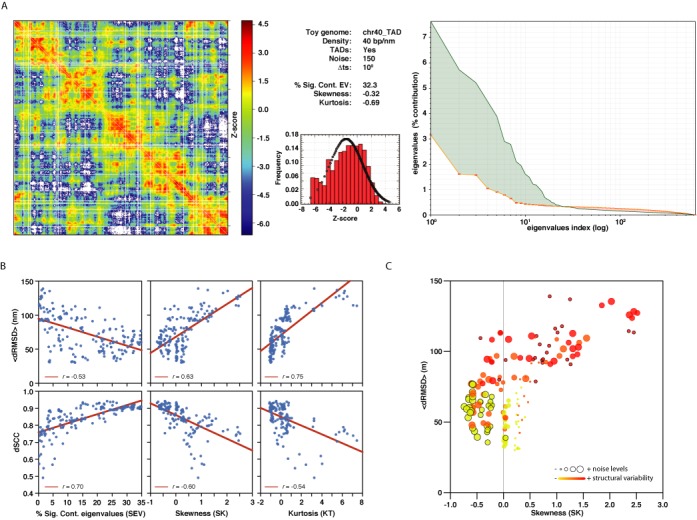
Matrix entropy. (**A**) Statistical measures for interaction matrix for chr40_TAD architecture. Simulated Hi-C matrix (left), statistical measures and Z-score distribution (middle) and eigenvalues plot (right). The eigenvalues plot shows the real distribution of values (green solid line) and the distribution for random matrices (orange solid line). The green area corresponds to the contribution of the significant eigenvalues (i.e. the SEV score). (**B**) Correlation of the statistical matrix properties (SEV, SK and KT) with the reconstructed model accuracy measures (dRMSD and dSCC). (**C**) Correlation plot between Skewness and dRMSD for the 168 simulated Hi-C matrices. Dots are colored with respect to the structural variability in the matrix (yellow to red for sets 0 to 6). The size of the dots is proportional to the level of noise in the matrix (small for α = 50 and large for α = 200). Highlighted red small dots indicate low noise and high structural variability matrices. Highlighted yellow large dots indicate high noise and low structural variability matrices.

To assess which features from the interactions matrices could be useful to predict the accuracy of the reconstructed models, we have calculated three statistical measures from the simulated Hi-C matrices (Materials and Methods). In particular we measure the contribution of the SEV from the matrix (SEV), the skewness (SK) and the kurtosis (KT) of the distribution of Z-scores. These three measures are indicative of the internal correlations in the matrix (SEV) and the deviation from normality of the distribution of interaction counts (SK and KT). These features are relevant for the modeling with the TADbit protocol since they determine the quantity and quality of the imposed restraints during modeling (9). In principle, an input matrix with high contribution of the SEV, skewness close to zero and low negative kurtosis is optimal for 3D reconstruction. For example, the toy genome architecture chr40_TAD, which results in accurate 3D reconstructed models (dRMSD = 47.2 nm and dSCC = 0.91), has a SEV of 32.3%, a SK of −0.32 and a KT of −0.69 (Figure 4A). Indeed, the three statistical measures from the simulated Hi-C matrices correlate with the final accuracy of the reconstructed models (Figure 4B). dRMSD correlates with SEV, SK and KT with a −0.53, 0.63 and 0.75 regression coefficient, respectively. dSCC correlates with SEV, SK and KT with an 0.70, −0.60 and −0.54 regression coefficient, respectively. Moreover, we observed that the SK statistic, which measures whether a matrix has a Z-score distribution skewed toward positive or negative values, could be used to discern between matrices with high structural variability from those with high experimental noise (Figure 4C). All but one of the simulated Hi-C matrices with large noise content (α = 200) and low structural variability (set 0) result in negative values of SK score. Similarly, all but two of the simulated Hi-C matrices with low noise content (α = 50) and high structural variability (set 7) result in positive values of SK score. In summary, we introduced here three simple statistics from the Hi-C matrices that can help us assess the likeliness of an interaction matrix to result in accurate reconstructed models.

### The MMP score

**Figure 5.**
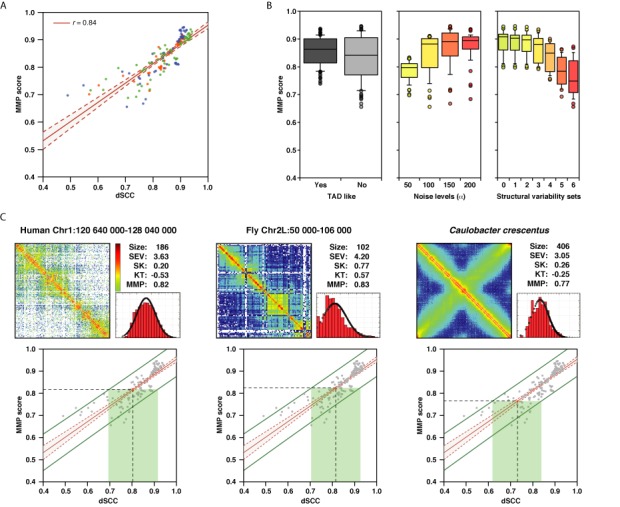
Predicting the accuracy of the reconstructed models. (**A**) Correlation between the MMP score and the dSCC accuracy measure. Points are colored by the density of the simulated Hi-C matrices (green 40, blue 75 and orange 150 bp/nm). Shaded area corresponds to the correlation confidence band. (**B**) MMP score distributions depending on genome architecture, noise and structural variability of the simulated Hi-C matrices. Panels from left to right show existence of TAD-like architecture, noise levels (yellow to red for α from 50 to 200) and structural variability (yellow to red from sets 0 to 6). (**C**) Example of MMP score and the predicted dSCC of the resulting models for genomic domains or entire genomes in real Hi-C matrices. For each panel we show the actual Hi-C matrix in Z-score scale (red to blue from positive to negative Z-scores), the four input statistics as well as the MMP score, the Z-score distribution shape and the predicted range of dSCC (green bar at 95% confidence level). Left panel for a genomic domain in chromosome 1 of the human genome (19), middle panel for a genomic domain in chromosome 2L of the fly genome (29) and right panel for the entire *Caulobacter crescentus* genome (30).

To assess whether we could *a priori* evaluate the adequacy of the input matrix for 3D reconstruction, we calculated a single score, here called MMP, combining four measures from the interaction matrices: its size, SEV, SK and KT values. We trained a linear regression with the four measures for the 168 simulated Hi-C matrices to obtain a single score that correlates the most with the dSCC accuracy measure of the 168 reconstructed models. The training set contains, thus, a variety of resolutions, experimental noise and structural variability. Using a 10-fold cross-validation the MMP score resulted in a final correlation with the dSCC of the reconstructed models of 0.84 (Figure 5A). The mean absolute error of the MMP score in predicting the dSCC accuracy of the models is 3.1%, which provides a clear predictive power to the new score. Indeed, the MMP score behaves as expected (Figure 5B). Simulated matrices built from toy genomes with TAD-like structure results in higher MMP score, which also increases with a slight presence of noise in the matrix and is clearly affected by the increase of structural variability. In summary, combining the three statistical scores from the simulated Hi-C matrices as well as its size into a single MMP score provides a means to *a priori* evaluate the modeling potential of the matrix. Matrices with high MMP scores are likely to result in accurate 3D reconstructed models.

To test the applicability of our new score, we selected three datasets of real Hi-C experimental data from human (19), fly (29) and bacterial (30) genomes and calculated their MMP score (Figure 5C). Of the three example matrices, the human genomic domain results in an MMP score of 0.82, which predicts a dSCC of 0.81 (0.69–0.92 at 95% confidence range). The best individual score for the human genomic domain is the skewness of the distribution, which approximates zero (SK = 0.20). However, the contribution of the significant eigenvalues is small (SEV = 3.61). Similarly, the *Caulobacter crescentus* genome matrix has good SK and KT values but poor SEV (0.26, −0.25 and 3.05, respectively). The resulting MMP score is 0.77, which predicts a dSCC of 0.73 (0.62–0.85 at 95% confidence range). Finally, the fly genomic domain is the one with the best MMP score (0.83) of the three real Hi-C matrices, which resulted in a predicted dSCC of 0.83 (0.72–0.94 at 95% confidence range). This result shows that, at different levels of predicted accuracy, real Hi-C matrices could be used in TADbit for 3D reconstruction of genomes and genomic domains.

## DISCUSSION

Recently, chromatin interaction matrices from 3C-based experiments have been used for modeling the 3D organization of genomes and genomic domains (5). Those approaches aim at providing a 3D representation of the bi-dimensional interaction matrices that can be explored to extract biological insights. Here, we have introduced a comprehensive analysis of the limitations of chromatin model building using a restraint-based mean-field approach. To do so, we have derived a series of simulated Hi-C matrices where the genomic architectures are pre-defined and the amount of noise and structural variability is controlled. The entire set of 168 simulated Hi-C matrices can be considered as a benchmark set for assessing the future developments of restraint-based methods for modeling genomes and genomic domains. To our knowledge, this is the first fully available dataset for benchmarking reconstruction methods, which can be freely accessed here: http://www.3DGenomes.org/datasets.

In our analysis, a total of six different genomic architectures were benchmarked. Those varied the resolution (or genomic density) as well as the presence of locally compacted regions resembling TADs observed for many organisms (19,31–32). The overall accuracy of the reconstructed models points to three main conclusions. First, independently of the genomic architecture, restraint-based mean-field modeling can provide accurate models with dRMSD as low as 30 nm and dSCC as high as 0.99 with the major variability in accuracy originating from the structural variability in the input matrices. Second, an increase of the matrix resolution (that is, low-density models with larger proportion of restraints per particle) results in more accurate reconstructed models. Therefore, increasing the sequencing depth of a Hi-C experiment will result not only in higher-resolution models (i.e. more bins in the interaction matrix) but also in models of higher overall accuracy. And third, the presence of a TAD-like architecture results in more accurate models at any levels of noise and structural variability. This increased accuracy can be interpreted as the result of a sharper structuring of the input Hi-C matrix for scales equal or larger than that of TADs, which are expected to be found under the form of compact globules (32). In vertebrates, these globules are believed to be the result of multiple specific chromatin loops induced by the bridging of several protein complexes, with CTCF as a major factor (6,33). Indeed, such specific loops can be easily integrated in polymer models of chromosomes (34) and should facilitate the inner reconstruction of TADs.

Typically, Hi-C experiments capture a limited number of all possible interactions in each cell (25,35) and thus are performed on a population of tens of millions of cells (8). This results in interaction matrices that have two main sources of variability originating from noise in the experiment and/or the natural conformational differences between genomes in each cell. Here we have simulated these two sources of variability by first varying the probability of capturing an interaction from the toy models (experimental noise) and second by deriving simulated interaction matrices from models of varying structural similarity. The results of our test clearly indicate that restraint-based mean-field reconstruction is robust to experimental noise but sensitive to high levels of structural variability. Indeed, at all levels of experimental noise, our method was able to reconstruct accurate models when structural variability was low. However, the reconstruction of models degraded significantly when the level of structural variability was high, indicating that mean-field methods may have difficulties capturing the entire structural diversity of the input matrices. It is important to note that our simulated Hi-C matrices with high levels of structural variability (set 6) contain homogenous structural variability where each of the toy structures can be considered as a ‘single cell state’ that is equally different to all other structures in the set. Despite these limitations, our approach was also able to capture part of the structural variability in the original set. Altogether, our results conclude that Hi-C interaction matrices from as homogenous as possible population of cells (e.g. synchronized in cell cycle, same cell state, unique cell type, etc.) are more adequate for 3D reconstruction. Interestingly, we also show that experimental noise, which could originate from limitations in any of the four main steps in 3C-based methods (that is, cell fixation, DNA fragmentation, DNA ligation and read-out by sequencing), is not highly relevant for 3D reconstruction.

Most of the reconstruction approaches, either those mean-field or population-based approaches, have a scoring function to minimize. The specific scoring function varies between methods but all aim at correlating the observed 3C-based interactions with those obtained from the reconstructing models. In our approach we find optimal parameters for the simulation by maximizing the Spearman correlation coefficient (TADbit-SCC) between the input interaction matrix and a contact map obtain from the models. We have shown here that this scoring function is appropriate for 3D reconstruction and that high TADbit-SCC result in accurate models, which validates our protocol for 3D reconstruction by TADbit. In practice, with our method the TADbit-SCC can be taken as a proxy of model accuracy. Additionally, we also provide, for the first time, a single measure (the MMP) calculated from the interaction matrix that highly correlates with the accuracy of the resulting models (*r* = 0.84, *P*-value < 0.001). The MMP score is composed of a weighted sum of four properties of the matrix (that is, its size, the percentage of contribution of significant eigenvalues in the interaction matrix as well as the skewness and kurtosis of the distribution of Z-scores in the interaction matrix). Interestingly, the skewness of the distribution has an additional property that allowed us to differentiate between matrices rich in experimental noise from those high in structural variability. Negative skewness matrices (that is, with a long positive tale) are likely to contain a large proportion of experimental noise. Positive skewness matrices (that is, with a long negative tail) are likely to be obtained from a population of cells with large structural variability. We applied our new MMP score to three published Hi-C interaction matrices. The results indicate that the 3D reconstruction of two genomic domains from the human and fly datasets as well as the entire *C. crescentus* genome could result in accurate models.

In summary, we provide a dataset of simulated toy structures and their respective Hi-C matrices that can be used for benchmarking restraint-based methods for 3D reconstruction. Our dataset was used to show that such methods are adequate for building 3D models of genomes and genomic domains. Moreover, we have shown that these methods are robust with respect to experimental noise but are more sensitive to structural variability in the input matrices. Experimentalists aiming to generate 3C-based interaction matrices for 3D reconstruction are thus encouraged to obtain the most homogenous cell population before performing the experiments. Finally, we provide for the first time a new score (here called MMP score) that allows predicting *a priori* the accuracy of the resulting models by calculating a limited number of properties of the input interaction matrices. Such score may probe very useful for defining whether a newly generated interaction matrix can be useful for obtaining accurate 3D models, which can then be more easily explored to extract biological insights.

## AVAILABILITY

The initial structural sets for the six tested toy genome architectures, their derived interaction matrices and the reconstructed 3D models are available at http://www.3DGenomes.org/datasets.
